# Deacetylation of Histones and Non-histone Proteins in Inflammatory Diseases and Cancer Therapeutic Potential of Histone Deacetylase Inhibitors

**DOI:** 10.2174/0113892029265046231011100327

**Published:** 2023-11-22

**Authors:** Ezgi Man, Serap Evran

**Affiliations:** 1 Department of Biochemistry, Faculty of Science, Ege University, 35100, İzmir, Türkiye;; 2 EGE SCIENCE PRO Scientific Research Inc., Ege University, IdeEGE Technology Development Zone, 35100, Bornova-Izmir, Türkiye

**Keywords:** Autoimmune diseases, epigenetics, histone protein modifications, non-histone protein modifications, HDACs, HDAC inhibitors, lysine deacetylation, histone deacetylase

## Abstract

Epigenetic changes play an important role in the pathophysiology of autoimmune diseases such as allergic asthma, multiple sclerosis, lung diseases, diabetes, cystic fibrosis, atherosclerosis, rheumatoid arthritis, and COVID-19. There are three main classes of epigenetic alterations: post-translational modifications of histone proteins, control by non-coding RNA and DNA methylation. Since histone modifications can directly affect chromatin structure and accessibility, they can regulate gene expression levels. Abnormal expression and activity of histone deacetylases (HDACs) have been reported in immune mediated diseases. Increased acetylated levels of lysine residues have been suggested to be related to the overexpression of inflammatory genes. This review focuses on the effect of HDAC modifications on histone and non–histone proteins in autoimmune diseases. Furthermore, we discuss the potential therapeutic effect of HDAC inhibitors (HDACi) used in these diseases.

## 1 INTRODUCTION

**Fig. (1):**
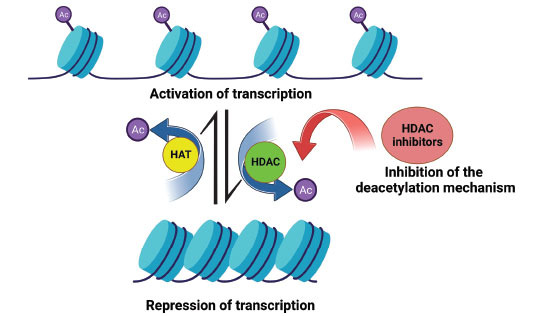
Histone acetylation and deacetylation regulation mechanism. Histone acetyl transferases (HATs) catalyze the transfer of acetyl groups, histone deacetylases (HDACs) remove the acetyl groups from the lysine residues. (Created with BioRender.com).

**Fig. (2):**
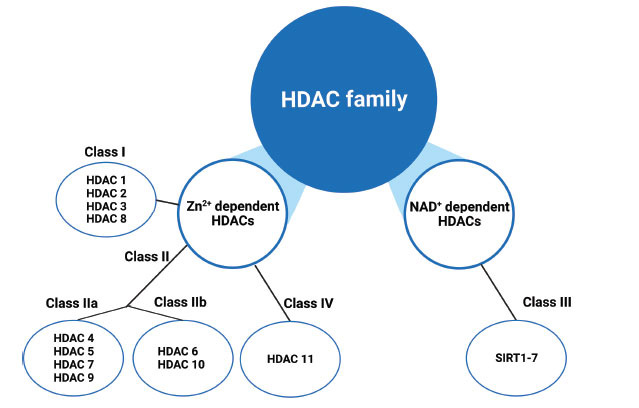
Classification of histone deacetylase (HDAC) family (Created with BioRender.com).

Post-translational modifications (PTMs) include methylation, glycosylation, ubiquitination, acetylation, phosphorylation and nitrosylation [1]. PTMs alter protein functions by regulating their stability and activity [2]. Epigenetic modifications involve many different cellular signalling pathways and contribute to various human diseases' pathogenesis [3]. Reversible lysine acetylation of histones is one of the important mechanisms for controlling gene expression, inflammation, cell development and differentiation [4]. Lysine acetylation levels are mediated by two enzyme groups: histone deacetylases (HDACs) and histone acetyltransferases (HATs) (Fig. **1**) [5]. Furthermore, HDACs and HATs also target many non-histone substrates. This suggests that lysine-acetylation is also critical in the cell proteome and protein function beyond chromatin accessibility mediated gene regulation [6]. Accumulating evidence shows that abnormal activities of HDACs and HATs activities play a crucial role in inflammatory diseases [7]. HDAC catalysis removes acetyl groups from lysine residues on histone protein tails. Mammalian HDACs family consists of 18 members, and they are divided into Class I (HDAC1, HDAC2, HDAC3 and HDAC8), Class IIa (HDAC4, HDAC5, HDAC7 and HDAC9), Class IIb (HDAC6 and HDAC10), Class III (SIRT1-7) and Class IV (HDAC11) (Fig. **2**) [8]. Classes I, II and IV have highly conserved domains and their deacetylation activity is Zn^2+^ dependent, while Class III enzyme sub-group deacetylase activity is NAD^+^ dependent [9]. HDACi can be used to inhibit HDAC activity in diseases such as cancer, immune diseases, neurodegenerative diseases, diabetes and cystic fibrosis [10]. HDAC inhibitors can be categorized according to their synthetic or natural composition, subclass-class specificity, and chemical types of structures. Generally, they are divided into two classes: HDAC-pan inhibitors and HDAC-specific inhibitors [11]. HDAC inhibitors are grouped into four main subgroups based on their chemical composition: hydroxamates, benzamides, cyclic tetrapeptides and short-chain fatty acids. HDAC inhibitors are considered novel epigenetic drugs, and their therapeutic potential is widely tested in various disease models [12].

## 2 CYSTIC FIBROSIS

Cystic fibrosis is a genetic disorder caused by mutations in the cystic fibrosis transmembrane conductance regulator (CFTR) gene [13]. CTFR protein is responsible for the transport of chloride and bicarbonate ions, and mutations that impair its function favor lung infection by opportunistic pathogens, *Pseudomonas aeruginosa* being the major one [14]. Excessive inflammatory response to *P. aeruginosa* plays a critical role in lung damage [15-17]. Hence, anti-inflammatory agents are considered as a treatment option [18, 19]. HDAC inhibitors are among those agents with promising results for reducing the inflammation in cystic fibrosis. Suberoylanilide hydroxamic acid (SAHA), a pan-HDAC inhibitor, was shown to modulate the inflammation caused by *P. aeruginosa* lipopolysaccharide [20]. In this study, treatment of *Cftr*+/+ or *Cftr*−/− mice with SAHA resulted in changes in interleukin-6 (IL-6) levels, nuclear factor kappa B (NFκB)-mediated signaling and neutrophil chemotaxis/activation. In another study [21], the efficiency of HDAC6 inhibitor, which was identified by screening patents and research papers was evaluated in the mouse model of *P. aeruginosa* acute and chronic respiratory infection. It was shown that inhibition of HDAC activity resulted in the reduction of several inflammatory interleukins, chemokines, growth factors, and interferon gamma (IFN-γ). Another interesting study revealed how the quorum sensing molecule 2-aminoacetophenone secreted by *P. aeruginosa* could affect HDAC1-dependent chromatin modification [22]. In this study, 2-aminoacetophenone was found to be responsible for HDAC1-mediated deacetylation of histone 3 at lysine 18 (H3K18) at the promoter sites of the autophagy gene *Beclin*1 and the lipid biosynthesis gene *Scd*1, resulting in decreased expression of these genes. It was concluded that *P. aeruginosa* was able to reduce the macrophage activity by modulating membrane lipids and autophagy. In addition to their anti-inflammatory effects, HDAC inhibitors were also found to have repairing activity on loss-of-function CTFR mutants. The pan-HDAC inhibitor SAHA was shown to restore the surface channel activity of CTFR phenylalanine 508 deletion (F508 del) variant [23]. In the same study, silencing of both HDAC1 and HDAC7 was found to enhance the stability of CTFR mutant. In particular, silencing of HDAC7 was found to be more effective in terms of restoring channel activity. The authors proposed that HDAC inhibitors could exert their effect through altering the transcriptional level of CFTR-related genes or by altering post-translational acetylation levels of their non-histone substrates. In a similar study [24], HDAC inhibitors were shown to restore the transport function of the F508del variant of CTFR. In addition, a similar effect on the other CTFR variants were observed, albeit with different degrees of response to HDAC inhibitors. The authors concluded that HDAC inhibitors rescued CTRF trafficking through downregulation of HDAC7 and abrogation of the maladaptive stress response (MSR). In another study, depletion of HDAC6 in the cystic fibrosis mouse model was found to restore the aggressive inflammatory response back to to wild-type profiles [25].

## 3 RHEUMATOID ARTHRITIS

Rheumatoid arthritis is a chronic autoimmune disease with both environmental and genetic basis [26]. Cytokines [27] and chemokines [28] are mainly responsible for inflammation, which results in joint damage. Previous studies showed [29] that the inflammation mechanism involves multiple signal transduction pathways regulated through epigenetic mechanisms. For instance, HDAC3 was shown to be involved in type-I interferon (IFN) production and regulation of inflammatory gene expression [30]. Another study [31] showed that the overexpressed HDAC2 in rheumatoid arthritis synovial tissue regulated the signalling pathway of the inflammatory mediator chemokine CC motif ligand 7 (CCL7). Similarly, HDAC6 was shown to be overexpressed in the synovium tissues of the mouse model and activated the nuclear factor-kappaB (NF-kappaB) signalling pathway by deacetylating its non-histone protein substrate myeloid differentiation primary response 88 (MyD88) [32]. Similarly, another study revealed that non-histone proteins were among the substrates of HDAC6 [33]. It was shown that inhibition of HDAC6 resulted in hyperacetylation of cytoskeletal proteins tubulin and cortactin. In addition, it decreased the production of interleukin-6 (IL-6) and the matrix metalloproteinases MMP1 and MMP3, thereby suppressing inflammation. Those studies proposed HDAC inhibitors as anti-inflammatory agents for treating rheumatoid arthritis [34]. For instance, the selective HDAC6 inhibitor CKD-506 was shown to prevent experimental arthritis in a murine model [35]. M-134, another HDAC6-selective inhibitor, was shown to reduce the level of chemokine (C-X-C motif) ligand 10 (IP-10), interleukin-17A (IL-17A), and tumour necrosis factor-alpha (TNF-α) expression. Moreover, a combination of M-134 and the drug tofacitinib enhanced the expression of different cytokines, adhesion factors and chemokines involved in immune cell migration and chemoattraction [36]. The selective HDAC1-inhibitor TTA03-107 was shown to suppress the production of inflammatory cytokines and reduce the severity of autoimmune arthritis [37]. As reviewed elsewhere [38], a combination of HDAC, inosine monophosphate dehydrogenase (IMPDH), mammalian target of rapamycin (mTOR), and Janus kinase (JAK) inhibitors could be promising to reduce the inflammation caused by increased cytokine levels. However, it should not be overlooked that inhibition of some HDAC isoenzymes may not yield the desired anti-inflammatory effects [39]. For instance, the inflammatory stimuli were shown to suppress HDAC5 expression. Moreover, the silencing of HDAC5 increased the levels of different chemokines and cytokines [40].

## 4 ATHEROSCLEROSIS

Atherosclerosis is a chronic inflammatory disease characterized by plaque formation in the walls of arteries and leading to cardiovascular disease and stroke [41]. Several studies revealed the connection between inflammation and atherosclerosis [42-45]. The impact of epigenetic mechanisms, such as DNA methylation, histone methylation, and acetylation of histone and non-histone proteins, on the polarization of macrophages was revealed [46]. HDAC isoenzymes are involved in endothelial dysfunction through different mechanisms [47]. In particular, inflammatory factors are activated by HDACs, *via* histone acetylation [48, 49]. A remarkable issue is that not all HDAC isoenzymes show disease-inducing effects [50]. For instance, HDAC7, associated with myocyte enhancer factor-2 (MEF2), was shown to protect endothelial integrity by downregulating matrix metalloproteinase MMP10 gene transcription [51]. In contrast, other isoenzymes such as HDAC3 [52], HDAC6 [53] and HDAC9 [54] were shown to contribute to the development of atherosclerosis. Hence, HDAC inhibitors were proven to be effective in reducing inflammation [55].

## 5 COVID-19

Excessive inflammatory response and cytokine storm play critical roles in pathogenesis and severity of the coronavirus disease 2019 (COVID-19) [56-58]. Hence, the effectiveness of anti-inflammatory drugs was intensively investigated [59-62]. Considering the inflammatory roles and the link between COVID-19 and epigenetic mechanisms [63-66], HDACs and HDAC inhibitors were also under investigation. For instance, several HDAC6 inhibitors were tested on the *in vitro* models of immune and epithelial cells by mimicking the cellular status after viral infection [67]. The authors showed that pro-inflammatory cytokines and interferon pathway genes were downregulated. In addition, the HDAC6 selective inhibitor ITF3756 was capable of upregulating the genes responsible for T-cell memory phenotypes. A screening study of the clinically approved HDAC inhibitors showed that romidepsin, panobinostat, givinostat hydrochloride monohydrate, CAY10603, and sirtinol were able to inhibit the cellular entry of COVID-19 [68]. A further study showed that the HDAC inhibitor panobinostat suppressed the expression of angiotensin-converting enzyme 2 (ACE2) receptor in the gastric adenocarcinoma cell line [69]. In another study, a similar result was obtained and valproic acid was shown to reduce the expression of angiotensin-converting enzyme 2 (ACE2) and neuropilin-1 (NRP1) receptors [70]. HDAC inhibitors were also proposed as neuroprotective agents against COVID-19 infection, mainly through downregulation of proinflammatory cytokines [71].

## 6 ASTHMA

Asthma is a chronic disease defined by airway inflammation, hyperresponsiveness, increased mucus secretion and remodelling [72]. Asthmatic inflammation is classified into 4 different groups; paucigranulacytic, eosinophilic, neutrophilic and mixed granulocytic. Airway inflammation has similar symptoms, like breathlessness, cough, wheezing, chest tightness, and dyspnoea [73]. Numerous asthma cases have reported increased interleukin-4 (IL-4) and interleukin-5 (IL-5 levels, eosinophils-mediated infiltration and activated mast cells. Glucocorticoids are used as the main therapy agent for asthma [74]. However, they cause undesired side effects. In addition, mixed granulocytic type of airway inflammation is unresponsive to standard/high-dose glucocorticoid treatment [75]. Therefore, alternative treatment approaches are needed [76]. HDAC2 enzyme activity alters chromatin structure and regulates inflammatory, anti-inflammatory gene expression in airway inflammation [77]. Various human asthma and murine models have been reported to decrease HDAC2 expression and specific enzyme activity [78]. Decreased HDAC2 expression level is associated with activation of NFκB signalling. Bruton’s tyrosine kinase (BTK) is expressed in both innate and adaptive immune cells such as neutrophils, B cells and macrophages. An earlier study showed that inhibition of BTK by Inrutinib was effective in mouse models of eosinophilic and neutrophilic airway inflammation [79]. Another study combined dexamethasone (corticosteroid) therapy and BTK inhibitor ibrutinib to test their therapeutic effects in cockroach allergen extract (CE)-induced mixed granulocytic inflammation mice model. Corticosteroids were found to downregulate the pulmonary inflammation-related gene expressions, such as tumour necrosis factor-alpha (TNF-α), interleukin-8 (IL-8), granulocyte-macrophage colony-stimulating factor (GM-CSF), inducible nitric oxide synthase (iNOS), interleukin-1β(IL-1β) and monocyte chemoattractant protein 1 (MCP1). Decreased HDAC2 expression was related to increased inflammatory cytokines [80]. In this study, BTK inhibition by Ibrutinib reestablished HDAC2 expression level and reduced inflammatory cytokines and NFκB expression. This study suggested that regulation of HDAC2 expression level by BTK inhibition might be an alternative approach to obtaining sensitivity to corticosteroids in granulocytic asthma [81]. Numerous human health investigations have studied curcumin’s (CUR) biological potential. The studies showed that curcumin has anti-oxidant/inflammatory and anti-allergic properties, and it functions as a natural HDAC-pan inhibitor [82]. Butyrate is an HDAC inhibitor, and it suppresses IL-8 expression. In another study, sodium butyrate (SoB) and CUR were used for modulating structural changes in the mouse model of asthma. HDAC1 and HDAC3 were extensively related to allergic-induced asthma [83]. The research authors concluded that SoB and CUR-mediated inhibition could effectively restore structural changes in airways, also suppress HDAC1 and NF-kB. In conclusion, the therapeutic properties of HDACi have offered alternative treatments for different human diseases. There is a need for new research for a better understanding of inhibitor/pathway relations [84].

## 7 NEUROINFLAMMATION

Recent findings indicate that neuroinflammation plays a crucial role in a range of neurological conditions, encompassing central nervous system (CNS) traumas, depression, and neurodegenerative illnesses such as Alzheimer's and Parkinson's diseases [85]. Neurological disorders' severity can be mitigated by inhibiting neuroinflammation [86]. HDACs play a pivotal role in modulating immune responses and inflammatory processes. HDACi have emerged as a prominent area of interest in investigating anti-inflammatory pharmaceuticals [87]. Earlier studies showed that in situations of brain injury and neurodegenerative disease, it is typical to observe an overexpression of HDAC1 and HDAC2 in microglia. The phenomenon induces polarization of microglia towards M1 macrophage. It results in the release of a considerable quantity of inflammatory mediators, which may ensue from the deacetylation of signal transducer and activator of transcription (STAT1/3), thereby intensifying the activation of the NF-κB signalling cascade [88]. The activation of the NF-κB signalling pathway ultimately results in the activation of microglia, thereby intensifying neuroinflammation and increasing neuronal damage. The same study has demonstrated that the application of HDAC inhibitor SAHA can impede the M1-polarization of microglia, reduce neuroinflammation dependent on HDAC1/2, and protect neuronal cells [89]. In another study, pan-inhibitory valproic acid (VPA) has been used to regulate STAT1/NF-κB and JAK2 (Janus Kinase 3)/STAT3 signal pathways to control microglial function and suppress spinal neuroinflammation in neuropathic pain [90]. HDACII inhibitory Tubastatin A has been used in cerebral ischemia; it has increased regulatory T cell (Treg) immunosuppressive ability and regulated interleukin-10 (IL-10) expression levels [87]. Histone deacetylases (HDACs) regulate gene expression by deacetylating histones and related proteins [86]. Additionally, HDACs have been found to directly deacetylate molecules involved in inflammatory signalling pathways, regulate the activation of glial cells in the central nervous system, and promote neuronal survival [91]. Further clarification is needed regarding the precise mechanisms HDAC regulates neuroinflammation [92].

## 8 CANCER

HDACs play several roles in cancer cell metabolism and they regulate cell cycle, apoptosis, DNA-damage response, metastasis, angiogenesis, autophagy [93-95]. Hence, dysregulation of HDACs results in cancer initiation and progression [96]. As reviewed elsewhere [97], HDAC8 is overexpressed in different types of cancers and the level of overexpression is correlated with the advanced stage of breast cancer and neuroblastoma. Similarly, HDAC2, HDAC3 and HDAC6 are overexpressed in lung cancer [97], human cholangiocarcinoma [98], and colon cancer [99], respectively. In addition, the overexpressed class I HDACs have been shown to promote drug resistance in glioma cells [100]. Because of the crucial roles of HDACs in cancer, HDAC inhibitors (Table **1**) have been proposed as anti-cancer agents [101-105]. For instance, the class I HDAC inhibitor valproic acid has been shown to enhance the effectiveness of chemotherapy agents in human melanoma cells [106]. Similarly, the class I HDAC inhibitor domatinostat has been shown to sensitize pancreatic cancer cells to chemotherapy by exerting its effect on the transcription factor FOXM1 [107]. Likewise, the potential of HDAC inhibitors to overcome immunotherapy resistance has been revealed [108]. There is also a growing interest in dual HDAC inhibitors targeting both HDAC and another cancer target, such as phosphoinositide 3-kinases [109], microtubule polymerization [110], bromodomain and extra-terminal [111]. As reviewed elsewhere [112], HDAC-based dual drugs have been proposed to be superior to single-targeted drugs in terms of therapeutic efficiency. Despite the great potential of HDAC inhibitors, it should not be overlooked that not all HDAC isozenzymes are related to cancer progression. A remarkable study has shown that pan-HDAC inhibitor promotes breast cancer metastasis due to the inhibition of HDAC4 [113]. Another study has revealed the tumor suppressive role of HDAC10 in cervical cancer [114].

## CONCLUSION

This mini-review summarized an overview of the latest literature on utilising HDAC inhibitors as pharmacological agents for the modulation of autoimmunity and inflammation. The summary of this review and outcomes from numerous investigations on autoimmune and autoinflammatory disorders clearly suggest that HDAC inhibitors have significant therapeutic potential in controlling the symptoms of immune-mediated diseases. Developing isoform-specific HDAC inhibitors is essential for effectively treating autoimmune disorders while overcoming adverse effects. In conclusion, a better understanding of the molecular consequences of HDAC inhibition is required to develop alternative treatment strategies for autoimmune diseases. Corepressor complexes consist of a variety of proteins that play a role in the repression of transcription. These proteins include DNA-binding proteins, histone deacetylases (HDACs), and components involved in the structural organization of chromatin. The role of corepressor function is crucial in controlling an extensive range of biological processes, including development, differentiation, and signal transduction. HDAC1, HDAC2, and HDAC3 generally function as a corepressor complex in transcriptional regulation. HDACs acting on both histone and non-histone proteins are attractive drug targets in a wide range of diseases. Hence, there is much interest in the discovery of HDAC inhibitors. However, the major limitation is that all of the FDA-approved drugs are pan-inibitors with no HDAC isoenzyme selectivity. Considering that each HDAC isoenzyme may have counter effects on the disease mechanism of interest, the design of isoenzyme-specific inhibitors is critical to prevent off-target effects and toxicity. Another issue is that HDACs are not only effective on histone proteins but also non-histone proteins. Although the number of studies on distinct biological functions of HDACs increases by year, there are still unknowns about the non-histone substrates, as well as the interaction partners of HDAC isoezymes. As more structural and mechanistic information is gathered, the therapeutic potential of HDACs is expected to be increased in the future. The interest in combination therapy approaches, as well as in dual-inhbitor design is encouraging efforts for the field of HDAC inhibitor research.

## Figures and Tables

**Table 1:** HDACi classification.

**HDAC Inhibitors**	**Types of HDAC Inhibitors**
MS-275 (Entinostat)	Benzamide
Apicidin Depsipeptide Trapoxin A	Cyclic peptide
Valproic acid Butyrate	Short chain fatty acid
Tubacin Belinostat Vorinostat (SAHA)	Hydroxamate

## LIST OF ABBREVIATIONS

ACE2: Angiotensin-converting Enzyme 2
BTK: Bruton’s Tyrosine Kinase
CFTR: Cystic Fibrosis Transmembrane Conductance Regulator
CNS: Central Nervous System
COVID-19: Coronavirus Disease 2019
CUR: Curcumin’s
GM-CSF: Granulocyte-macrophage Colony-stimula- ting factor
HATs: Histone Acetyltransferases
HDACi: HDAC Inhibitors
HDACs: Histone Deacetylases
IFN-γ: Interferon gamma
IL-6: Interleukin-6
IMPDH: Inosine Monophosphate Dehydrogenase
iNOS: inducible Nitric Oxide Synthase
JAK: Janus kinase
MCP1: Monocyte Chemoattractant Protein 1
MEF2: Myocyte Enhancer Factor-2
MSR: Maladaptive Stress Response
mTOR: mammalian Target of Rapamycin
NFκB: Nuclear Factor Kappa B
NRP1: Neuropilin-1
PTMs: Post-translational Modifications
SAHA: Suberoylanilide Hydroxamic Acid
SoB: Sodium Butyrate
TNF-α: Tumour Necrosis Factor-alpha
VPA: Valproic Acid
